# Propensity score matching analysis comparing outcomes between primary and revision Roux-en-Y gastric bypass after adjustable gastric banding: a retrospective record-based cohort study

**DOI:** 10.1007/s00464-022-09675-z

**Published:** 2022-10-05

**Authors:** Mohamed Hany, Iman El Sayed, Ahmed Zidan, Mohamed Ibrahim, Ann Samy Shafiq Agayby, Bart Torensma

**Affiliations:** 1 Department of Surgery, Medical Research Institute, Alexandria University, 165 Horreya Avenue, Hadara, Alexandria, 21561 Egypt; 2 Biomedical Informatics and Medical Statistics Department, Medical Research Institute, Alexandria University, Alexandria, Egypt; 3 Clinical Epidemiologist, Leiden University Medical Center (LUMC), Leiden, The Netherlands

**Keywords:** Primary Roux-en-Y gastric bypass, Revision Roux-en-Y gastric bypass, Adjustable gastric band, Bariatric surgery, Food tolerance

## Abstract

**Background:**

One-stage revision Roux-en-Y gastric bypass (RRYGB) after Laparoscopic adjustable gastric banding (LAGB) is widely adopted, but its safety is still debated.

**Objective:**

This study aimed to compare outcomes between primary Roux-en-Y gastric bypass (PRYGB and RRYGB after LAGB.

**Method:**

A retrospective record-based cohort study of patients who underwent PRYGB and RRYGB for failed LAGB and completed at least 2 years of follow-up from 2008 to 2019. Propensity score matching (PSM) analysis was conducted to obtain a balanced sample of patients with RRYGB and PRYGB interventions by adjusting for baseline covariates including age and sex.

**Results:**

Patients with PRYGB (*n* = 558) and RRYGB (*n* = 156) were included. PSM identified 98 patients for RRYGB and 98 patients for PRYGB. Both cohorts exhibited significant reductions in BMI compared to baseline values (*p* < 0.001), but reductions were significantly higher in PRYGB compared to those in RRGYB at 6 months (− 10.55 ± 8.54 vs. − 8.38 ± 5.07; *p* = 0.032), 1-year (− 21.50 ± 8.19 vs. 16.14 ± 6.93; *p* < 0.001), and 2 years (− 24.02 ± 7.85 vs. − 18.93 ± 6.80; *p* < 0.001), respectively. A significant improvement in food tolerance from the 1st to the 2nd year was seen after RYGB (*p* < 0.001). The rates of early and late complications were similar in both cohorts (*p* = 0.537, *p* = 1.00). Overall re-intervention rates were 5.1 and 3.1% for RRYGB and PRYGB *p* = 0.721). Both cohorts exhibited significant improvement in comorbidities after 2 years (*p* < 0.001).

**Conclusions:**

One-stage RRYGB for failed LAGB is safe and effective with comparable rates of complications, re-interventions, and resolution of associated comorbid conditions compared to PRYGB.

Laparoscopic adjustable gastric banding (LAGB) gained considerable popularity in the early 2000s [1]. In 2011, LAGB was the second most commonly performed procedure in the USA after Roux-en-Y gastric bypass (RYGB) [2]. LAGB was a simple, easy-to-do, reversible, restrictive laparoscopic procedure with expected rapid weight loss and low morbidity [3, 4]. Unfortunately, there have been high failure rates regarding weight loss, whereby 30–50% of patients who underwent LAGB suffered from inadequate weight loss or weight regain and required a revision procedure within 7 years [4–10]. Combined with the fact that long-term weight loss was unsatisfactory, various complications as reflux esophagitis, esophageal dilatation, band slippage, and band erosions caused a considerable number of patients who underwent LAGB to seek corrective procedures [5–9]. Options for revision surgery usually included band removal, and conversion to laparoscopic RYGB or laparoscopic sleeve gastrectomy (LSG) [7]. RYGB has been endorsed by many authors as a safe and effective option for revision surgery after failed LAGB [4, 11–16]. Also, meta-analyses have shown the safety and effectiveness of RYGB after failed LAGB [7, 17–21].

Revision RYGB (RRYGB) can be performed using either a one- or two-stage approach; in the latter; band removal and the ensuing procedure are performed in different sessions. The one-stage approach could minimize episodes of anesthesia, prevent second hospital admission, and reduce subsequent costs. Several single- and multi-center studies, and meta-analyses have supported the safety of the one-stage approach [4, 12, 13, 15, 22, 23].

Although LAGB is not a popular procedure anymore, it is still being performed in some centers. LAGB formed 5% of the bariatric procedures reported in the IFSO Global Registry between 2014 and 2018 [24]. Also, LAGB formed 0.9% of all bariatric procedures in the USA in 2019 declining from 35.4% in 2011 [25]. The revision surgery after LAGB will remain an important topic of bariatric surgery for a while in the future, and more data about its outcomes should be helpful to guide surgeons to get the best outcomes. The present study aimed to report our experience in one-stage conversion of LAGB to RYGB and highlight the effectiveness and safety of one-step conversion compared to primary procedures. We evaluated short-term outcomes including weight loss, complications, food tolerance (FT), and resolution of associated medical conditions.

## Materials and method

This was a retrospective cohort study at three specialized bariatric centers. Records of patients from 2008 to 2019 were analyzed to identify patients who underwent primary RYGB (PRYGB) or single-stage RRYGB after failed LAGB and completed all follow-up visits to date. Records with a minimal follow-up of 2 years were included.

All patients provided written and oral informed consent to use their data in future research. All data were anonymized. The study conformed to the principles of the Declaration of Helsinki and was approved by the ethics committee of the Medical Research Institute.

### Study endpoints

Weight loss, food tolerance, complications, and resolution of associated medical conditions.

### Data collection

Demographic data of the patients and associated medical conditions at the time of RYGB and or LAGB, operative time, concomitant operative procedures, and duration of hospital stay were collected for all patients. Furthermore, the lowest body mass index (BMI) after LAGB, causes for revision, and the time between LAGB and RRYGB were recorded.

### Preoperative workup for revision cases

All revision cases underwent virtual gastroscopy using multi-detector computed tomography (MDCT) and routine upper gastrointestinal endoscopy. Food tolerance (FT) was evaluated using a one-page questionnaire divided into 4 sections, 3 of which were used to calculate the score: overall patient satisfaction with eating (score 1–5); tolerability to certain food types (score 0–16); and frequency of vomiting/regurgitation (score 0–6), with a total score between 1 and 27; higher scores indicate better food tolerance [26].

### Parameters measured during the follow-up period after RYGB

Body Mass Index (BMI), percentage excess BMI loss (% EBMIL) and FT assessment at 6 months, 1- and 2 years; early complications during the first 30 days following surgery, and late complications that occurred subsequently; reoperations and readmissions; endoscopic findings for patients who required endoscopic examination during the follow-up period; and resolution/improvement of associated medical conditions such as diabetes mellitus, dyslipidemia, and hypertension, were recorded at the 2-year follow-up.

### Statistical methods

We used descriptive and inferential statistics for the analyses. The data were first tested for normality using the Kolmogorov–Smirnov test, a quantile–quantile (QQ) plot, and Levene’s test. Categorical variables were expressed as *n* (%). Continuous normally distributed variables were expressed as means ± standard deviations, and non-normally distributed data as medians and interquartile ranges for skewed distributions. Categorical variables were tested using the Pearson’s chi-square test or Fisher’s exact test, when appropriate. Normally distributed continuous data were tested using the Student t-test and in case of skewed data, with the Mann–Whitney U-test.

A propensity score matching (PSM) analysis was conducted by nearest neighbor matching, ratio1:1 at caliper 1 to obtain a balanced sample of patients between both groups. Average propensity score was statistically compared by independent sample *t*-test and illustrated with a histogram plot to ensure balanced distribution of the propensity score and proposed confounders between both groups. No missing data were reported for any of the covariates involved in the PSM analysis [27].

A mixed-design repeated-measures analysis of variance (ANOVA) test was conducted, to evaluate the effect of postoperative time, the main effect of surgery whether RYGB post LAGB or primary RYGB, and to determine whether the interaction is present in the change pattern of BMI and %EBMIL during different postoperative periods between both groups.

A multiple linear regression model was conducted with the entering method to assess the independent contribution of type of surgery, adjusted for age, sex, and preoperative BMI, on BMI change at 6 months, 1-, and 2 years of postoperative follow-up as the outcome variable. Assumptions in terms of linearity by scatter plot; homoscedasticity and normality by residual plot, histogram, normal probability plot, and independence of errors by Durbin–Watson test were determined. General estimation equation analysis was performed to produce unbiased average estimates with 95% confidence intervals (95% CI) of BMI reduction among patients who underwent RYGB post LAGB intervention and standard PRYGB at 2 years postoperative follow-up adjusted for age, sex, and preoperative BMI. The significance level for baseline variables and multivariable regression analysis was set at *p* < 0.05. All statistical tests were conducted using IBM SPSS statistics (IBM SPSS Statistics for Windows, Version 28.0. Armonk: IBM Corp.) and R (Version 4.0.4). packages.

## Results

This retrospective cohort study analyzed data from 2008 to 2019. A total of 558 patients who underwent PRYGB completed 2 years of follow-up and 102 patients with RRYGB after LAGB completed 2 years of follow-up.

### Lost to follow-up patients

Between 2008 and 2016, 691 patients underwent PRYGB, 558 (80.8%) completed 2 years of follow-up and 133 (19.2%) were lost to follow-up. Between 2008 and 2019, 121 patients had revision RRYGB, 102 (84.3%) completed 2 years of follow-up and 19 (15.7%) were lost to follow-up.

### Baseline Characteristics (Table 1)

**Table 1:** Comparison of demographic, associated medical conditions, and preoperative anticoagulant intake between patients undergoing RRYGB post LAGB and patients undergoing PRYGB before and after PSM

	Before PSM		Sig	After PSM		Sig
	RRYGB post LAGB (*n* = 102)	PRYGB (*n* = 558)		RRYGB post LAGB (*n* = 98)	PRYGB (*n* = 98)	
Age (years)	43.3 ± 7.0	37.8 ± 11.1	< 0.001*	42.8 ± 6.6	41.9 ± 9.3	0.429
Female Sex, *n* (%)	89 (87.3%)	407 (72.9%)	0.002*	85 (86.7%)	85 (86.7%)	1
Weight (kg)	130.3 ± 23.8	133.0 ± 26.1	0.158	129.2 ± 22.5	132.0 ± 29.7	0.052
Height (m)	1.7 ± 0.1	1.7 ± 0.1	0.385	1.7 ± 0.1	1.7 ± 0.1	0.335
Preoperative BMI (kg/m^2^)	46.7 ± 8.0	47.6 ± 7.0	0.123	46.5 ± 7.6	48.6 ± 7.8	0.059
Duration of hospitalization (days)	2.03 ± 0.17	1.98 ± 0.13	0.002*	2.03 ± 0.17	1.99 ± 0.10	0.046*
Operative time (min)	160.78 ± 34.20	42.62 ± 10.30	< 0.001*	159.59 ± 34.31	42.04 ± 9.22	< 0.001*
Preoperative associated medical problems						
One or more associated medical condition	49 (48.0)	265 (47.5)	0.919	46 (46.9)	43 (43.9)	0.667
IHD	8 (7.8)	53 (9.5)	0.596	8 (8.2)	11 (11.2)	0.469
Sleep apnea	7 (6.9)	54 (9.7)	0.367	7 (7.1)	5 (5.1)	0.551
Dyslipidemia	37 (36.3)	213 (38.2)	0.716	35 (35.7)	34 (34.7)	0.881
DM	5 (4.9)	56 (10.0)	0.100	4 (4.1)	11 (11.2)	0.060
HTN	9 (8.8)	55 (9.9)	0.746	9 (9.2)	6 (6.1)	0.420
Renal insufficiency	3 (2.9)	16 (2.9)	0.967	3 (3.1)	0	0.081
Preoperative anticoagulant intake	3 (2.9)	0	< 0.001*	3 (3.1)	0	0.081

*PSM* propensity score matching by nearest neighboring method, ratio 1:1; *IHD* ischemic heart diseases; *DM* diabetes mellitus; *HTN* hypertension

Mean age was 43.3 ± 7.0 years in the RRYGB cohort, and 37.8 ± 11.1 years in the PRYGB cohort (*p* = 0.02). Women comprised 87.3% of RRYGB cohort and 72.9% of the PRYGB cohort (*p* < 0.001). The average duration of hospitalization for RRYGB and PRYGB was 2.03 ± 0.17 vs. 1.98 ± 0.13 days, respectively (*p* = 0.002). The operative time was longer in the RRYGB group compared to that in the PRYGB group (160.78 ± 34.20 vs. 42.62 ± 10.3 min, respectively (*p* < 0.001).

### Propensity score matching (PSM)

Preoperative BMI, having more than one associated medical problem, and having diabetes mellitus, did not differ initially between the groups; therefore, they were not included as covariates for PSM. In total, 98 patients with RRYGB as well as 98 patients with PRYGB were matched and had comparable confounders: age (*p* = 0.429), sex (*p* = 1.00), preoperative BMI (*p* = 0.059), and having more than one associated medical problem (*p* = 0.677) (Table 1).

**Fig. 1:**
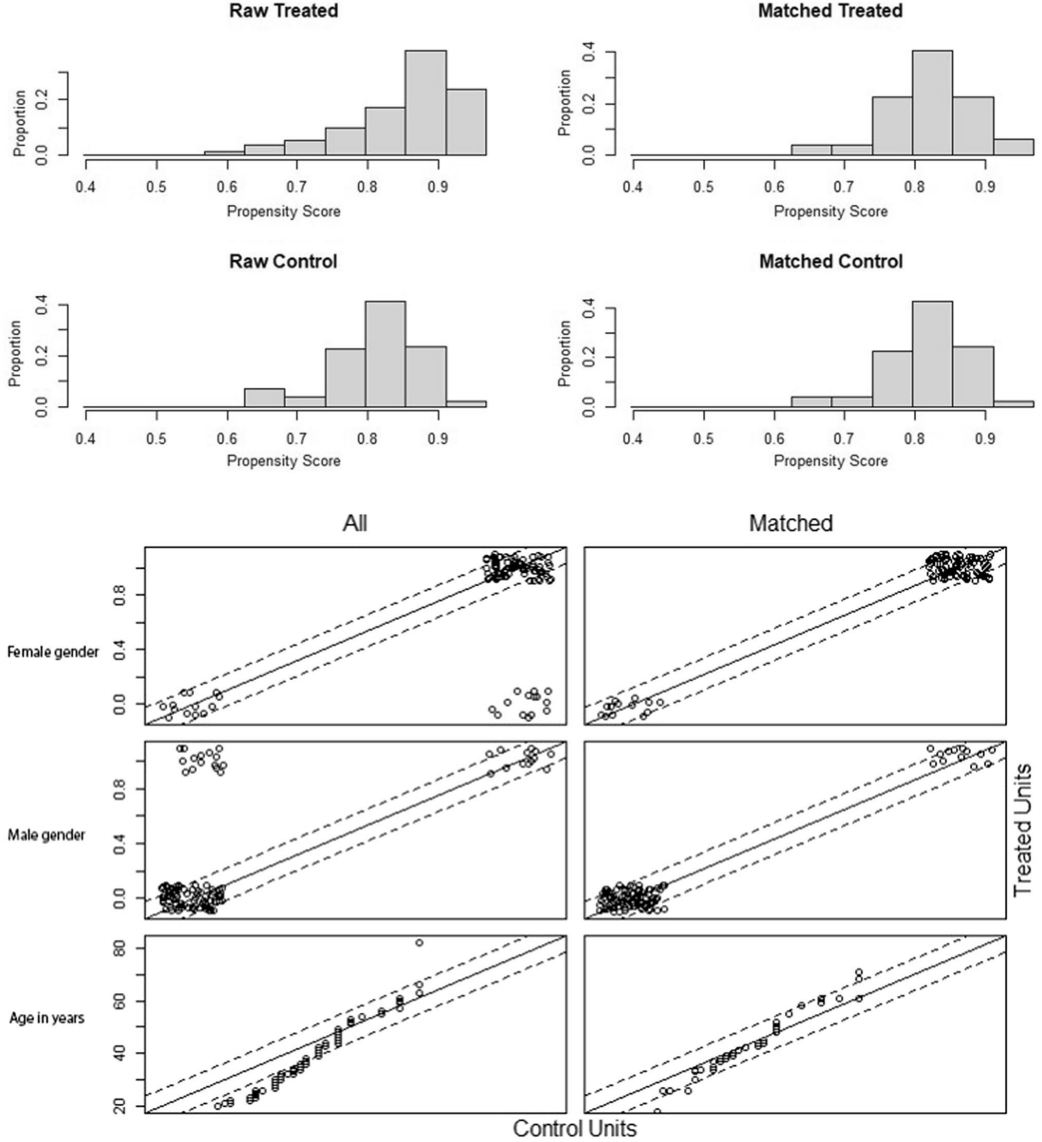
Propensity score matching (PSM)

A balanced distribution of propensity scores after matching between the groups showed a balanced distribution of age and sex covariates after matching for each intervention. The average propensity score was 0.6 ± 0.06 in RRYGB versus 0.6 ± 0.06 in PRYGB (*p* = 0.546) (Fig. 1).

### Preoperative findings in RRYGB cases

The main causes of revision where weight regain (74.4%) and insufficient weight loss (13.2%). The most common findings in the endoscopic examination were dilated esophagus (14.3%) and tight band (12.2%). Reflux esophagitis was seen in 9.18% of the cases [28]. Band erosion in the stomach was identified in one case (1.02%), (Appendix 1).

### BMI changes

After PSM, the average BMI before RYGB was 46.5 ± 7.6 and 48.6 ± 7.8 kg/m^2^ in the RRYGB and PRYGB cohorts, respectively; (*p* = 0.059). Two years following RYGB, the mean BMI was 27.54 ± 3.51 and 24.61 ± 3.15 kg/m^2^ for RRYGB and PRYGB cohorts, respectively. Both cohorts achieved significant reductions in BMI compared to the baseline values (*p* < 0.001) and between cohort’s delta − 2.93 after 2 years with a significantly lower BMI in the PRYGB group; (*p* = 0.007).

The reduction in BMI from baseline was significantly higher in the PRYGB group at 6 months (− 10.55 ± 8.54), 1 year (− 21.50 ± 8.19) and 2 years (− 24.02 ± 7.85) compared to those in the RRYGB group at 6 months (− 8.38 ± 5.07), 1 year (− 16.14 ± 6.93) and 2 years (− 18.93 ± 6.80) kg/m^2^ (*p* = 0.032, < 0.001, and < 0.001, respectively) (Table 2).

**Table 2:** Multivariate analysis and general estimation equation adjusted for age, sex, and BMI before RYGB and after PSM

Covariates	BMI change from baseline					
	Before PSM			After PSM		
	Coeff	[95% CI]	*p* value	Coeff	95% CI	*p* value
At 6 months^a^						
Primary RYGB	− 2.256	[− 3.89 to − 0.62]	0.007*	− 1.919	[− 3.648 to − 0.190]	0.030*
Male	− 0.546	[− 1.89 to .80]	0.426	.897	[− 1.762 to 3.556]	0.507
Age in years	− 0.079	[− 0.13 to − 0.02]	0.005*	− 0.154	[− 0.267 to − 0.040]	0.008*
Preoperative BMI ≥ 40	− 10.202	[− 11.86 to − 8.54]	< 0.001*	− 9.642	[− 12.114 to − 7.170]	< 0.001*
At 1 year^a^						
Primary RYGB	− 4.316	[− 5.799 to − 2.833]	< 0.001*	− 4.941	[− 6.741 to − 3.140]	< 0.001*
Male	− 0.188	[− 1.408 to 1.031]	0.762	2.220	[− 0.549 to 4.988]	0.115
Age in years	− 0.020	[− 0.070 to 0.029]	0.418	− 0.063	[− 0.181 to 0.054]	0.289
Preoperative BMI ≥ 40	− 10.933	[− 12.438 to − 0.429]	< 0.001*	− 11.710	[− 14.284 to − 9.136]	< 0.001*
At 2 years^a^						
Primary RYGB	− 3.793	[− 5.168 to − 2.417]	< 0.001*	− 4.655	[− 6.382 to − 2.927]	< 0.001*
Male	− 0.210	[− 1.341 to 0.921]	0.716	0.438	[− 2.218 to 3.095]	0.745
Age in years	− 0.020	[− 0.066 to 0.026]	0.385	− 0.049	[− 0.162 to .064]	0.397
Preoperative BMI ≥ 40	− 11.267	[− 12.663 to − 9.871]	< 0.001*	− 11.774	[− 14.244 to − 9.305]	< 0.001*

	Est. average	[95% CI]	Sig	Est. average	[95% CI]	Sig
General estimation equation regression analysis^b^						
Type of surgery						
RYGB post LAGB	− 10.948	[− 12.038 to − 9.858]	< 0.001*	− 10.289	[− 11.546 to − 9.033]	< 0.001*
Primary RYGB	− 14.403	[− 14.984 to − 13.823]		− 14.127	[− 15.589 to − 12.666]	
Preoperative BMI						
< 40	− 7.275	[− 8.164to − 6.387]	< 0.001*	− 6.687	[− 8.316 to − 5.059]	< 0.001*
≥ 40	− 18.076	[− 18.788 to − 17.36]		− 17.730	[− 18.853 to − 16.606]	

*p ≤ 0.05

^a^Data indicates change from baseline at 6 months, 1-, and 2 years. Coeff, Coefficients of the multiple linear regression analysis go along with 95% confidence intervals (CI), predictors are age, sex, preoperative BMI, and type of surgery

^b^General estimation equation to predict BMI change from baseline at 2 years postoperative follow-up adjusted for age, sex, preoperative BMI, and type of surgery

**Fig. 2:**
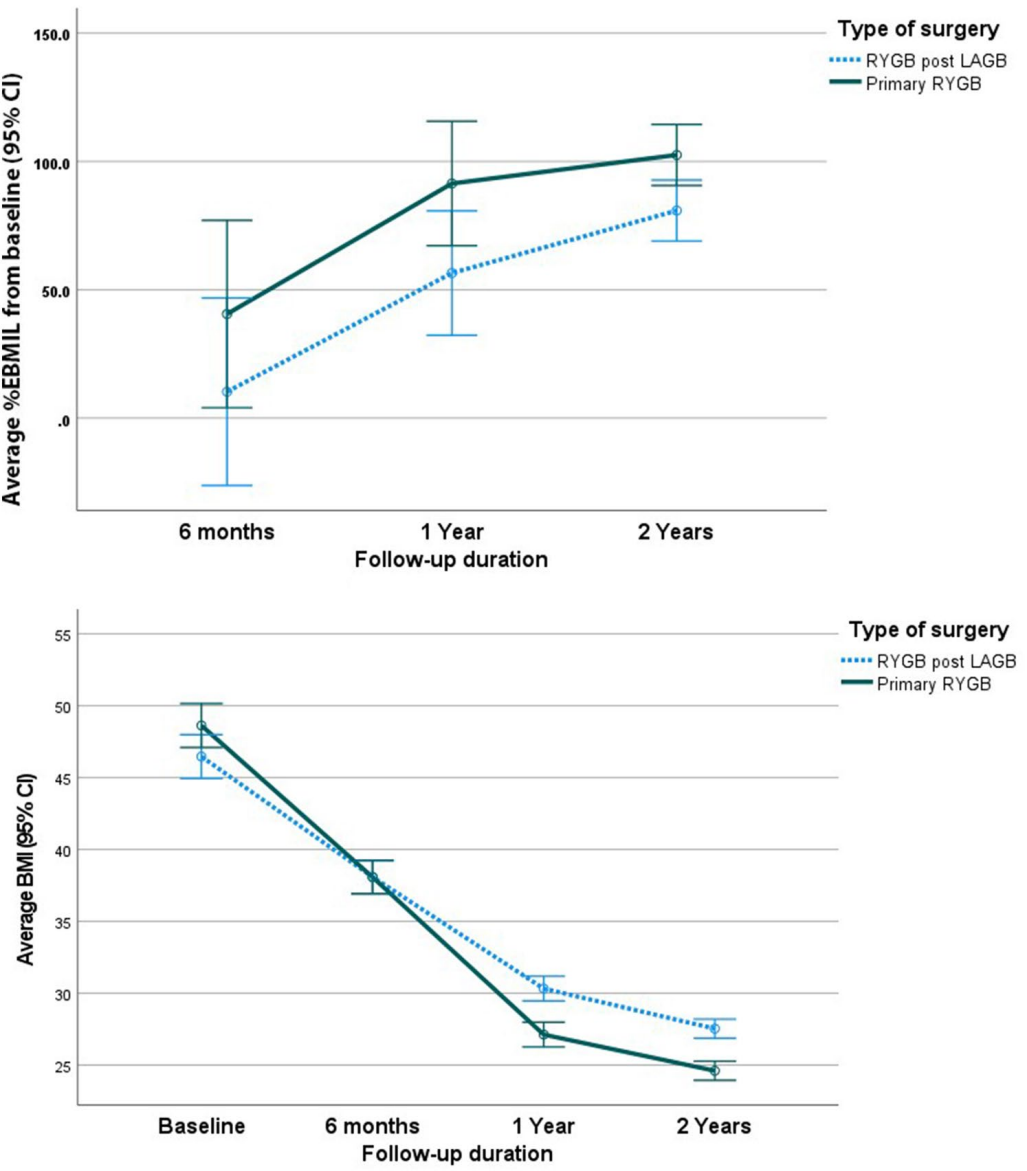
BMI changes after propensity score matching

The mixed-design ANOVA analysis revealed an overall significant decline in BMI at 6 months, 1- and 2 years postoperatively from baseline (*p* =  < 0.001). We detected the significant main effect of type of intervention with greater reduction of average BMI and greater increase in average %EBMIL among patients with PRYGB compared to RRYGB; *p* =  < 0.001 and 0.042 respectively. Significant interaction between type of surgery and changes in average BMI and average %EBMIL during the post-operative follow-up period accounted for the greater pattern of reduction in BMI and the greater increase pattern of %EBMI in PRYGB compared to RRYGB; *p* =  < 0.001 and *p* =  < 0.001, respectively; (Fig. 2).

### Multivariate analysis of BMI outcomes

After adjusting for age, sex, and preoperative BMI, patients who underwent PRYGB surgery experienced a significant reduction in BMI by 1.919 kg/m^2^ more than that of the RRYGB group at 6 months, 1 and 2 years postoperatively (after PSM − 1.919 kg/m^2^; 95% CI − 3.648 to − 0.190; *p* = 0.007), (after PSM − 4.941 kg/m^2^, 95% CI − 6.741 to − 3.140) and (after PSM − 4.655 kg/m^2^, 95% CI − 6.382 to − 2.927), respectively; (Table 2 and Fig. 2).

General estimation equation regression analysis revealed an estimated reduction of BMI from baseline by 10.289 kg/m^2^ (after PSM − 10.289; 95% CI − 11.546 to − 9.033) at 2 years postoperatively among patients who underwent RRYGB compared to an estimated reduction of BMI by 14.403 kg/m^2^ (after PSM − 14.127; 95% CI − 15.589 to − 12.666) for patients who underwent PRYGB.

Patients with preoperative BMI ≥ 40 experienced BMI reduction from baseline by 17.730 kg/m^2^ (after PSM − 17.730; 95% CI − 18.853 to − 16.606), whereas those with preoperative BMI < 40 experienced BMI reduction from baseline by 6.68 kg/m^2^ (after PSM − 6.687; 95% CI [− 8.316 to − 5.059) (*p* =  < 0.001) (Table 2).

### Food tolerance (FT)

**Fig. 3:**
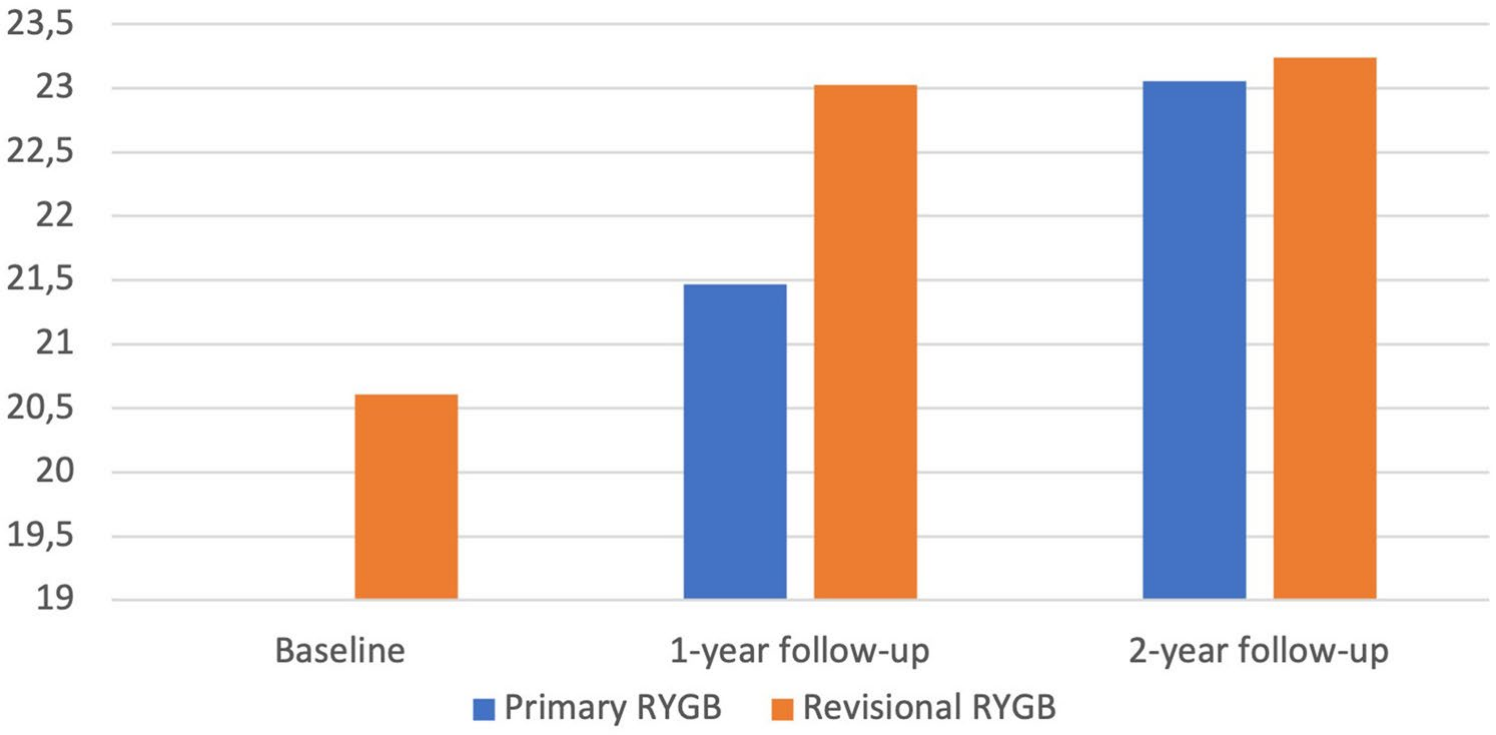
Food Tolerance (FT) at before surgery (baseline) and during follow-up after PRYGB and RRYGB

The mean FT score in the RRYGB cohort increased significantly from 20.61 ± 1.31 before RRYGB to 23.03 ± 0.84 and 23.24 ± 0.85 at 1 and 2 years following RYGB; (*p* < 0.001 and < 0.001, respectively). The mean FT score in the PRYGB cohort increased significantly from 21.47 ± 0.54 in the first year to 23.06 ± 0.79 in the second year; (*p* =  < 0.001). The RRYGB cohort exhibited a significantly higher FT score compared to that of the PRYGB in the first year of follow-up; (*p* =  < 0.001) (Fig. 3).

Mixed-design ANOVA analysis revealed an overall significant improvement in FT from the first to the second year after RYGB (*p* =  < 0.001). We detected a significant main effect of the type of intervention with a higher average FT change in PRYGB (*p* =  < 0.001). A significant interaction between change in average FT from the first to the second year after RYGB and type of surgery accounted for the greater pattern of improvement of FT in PRYGB compared to that of RRYGB (*p* < 0.001).

### Complications

The incidence of early complications was not significantly different in the PRYGB and RRYGB cohorts (*p* = 0.537). No incidence of leaks was recorded in either cohort. Intra-abdominal bleeding that mandated laparoscopic exploration due to hemodynamic instability occurred in two (2%) of the RRYGB patients. Thrombosis of the portal, mesenteric and splenic venous system (PMSVT) was recorded in three (3.1%) RRYGB patients and one (1%) PRYGB patient and presented at 15–24 days after surgery with persistent abdominal pain, low-grade fever and leukocytosis. The diagnosis was confirmed by MDCT with IV contrast. Two patients in the RRYGB cohort were managed conservatively with the use of anticoagulants and fluid resuscitation, whereas one RRYGB patient and the PRYGB patient required limited resection of a short segment of the jejunum.

Intestinal obstruction due to internal hernia through the mesenteric defect at the jejuno-jejunostomy was recorded in one (1%) patient in the RRYGB group, who presented 3 weeks after surgery, and was managed by laparoscopic exploration and repair. Melaena was encountered in three (3.1%) PRYGB patients and three (3.1%) RRYGB patients, and conservative management using IV fluids, blood transfusion, and discontinuation of enoxaparin was sufficient (Table 3).

**Table 3:** Comparison of outcomes (early, late complications, readmission, and reoperation) between RRYGB post LAGB and PRYGB groups after PSM

	RYGB post LAGB		Primary RYGB		Sig
	*n* (98)	%	*n* (98)	%	RR [95% CI]
Early complications					
No complications (ref)	87	88.8	93	94.9	^MC^p.537
MVO	3	3.1	1	1.0	3.13 [0.332 to 29.569]
Melena	3	3.1	3	3.1	1.07 [0.221 to 5.149]
Bleeding/abdominal	2	2.0	0	0.0	5.222 [0.254 to 107.286]
Intestinal obstruction/hernia	1	1.0	0	0.0	3.169 [0.131 to 76.763]
Wound infection	2	2.0	1	1.0	2.112 [0.195 to 22.89]
Late complications					
No complications (ref)	93	94.9	92	93.9	^MC^p1
Marginal ulcer/Melena	4	4.1	4	4.1	0.99 [0.255 to 3.844]
Port site hernia	1	1.0	2	2.0	0.50 [0.046 to 5.421]
Readmission					
No	92	93.9	95	96.9	^FE^p.497
Yes	6	6.1	3	3.1	2.0 [0.515 to 7.772]
Reoperation					
No	93	94.9	95	96.9	^FE^p.721
Yes	5	5.1	3	3.1	1.667 [0.409 to 6.784]

*RR* relative risk compares incidence of complication among patients undergoing RYGB post LAGB intervention relative to standard Primary RYGB standard approach, (95% CI): 95% Confidence interval, ref: reference category, *MC* Monte-Carlo significance, *FE* Fischer-Exact significance

The incidence of late complications was almost equal in the PRYGB and RRYGB cohorts (*p* = 1.00). Marginal ulcers were recorded, in four (4.1%) patients in each cohort. They presented with melena, epigastric pain, and vomiting. All eight patients were smokers, 2 were also on nonsteroidal anti-inflammatory drugs (NSAID). Medical treatment was successful in all patients. Port site hernias occurred in two (2%) patients in the RRYGB and one (1%) patient (0.72%) in the PRYGB group. All patients underwent surgical repair. Overall re-intervention rates were 5.1 and 3.1% for the RRYGB and PRYGB cohorts, respectively (*p* = 0.721). Readmissions were recorded in six (6.1%) patients in the RRYGB cohort and three (3.1%) patients in the PRYGB cohort (*p* = 0.497). The main reasons for readmission were melena, Portomesentric and splenic vein thrombosis (PMSVT), vomiting, and dehydration. (Table 3).

### Associated medical problems

Both the RRYGB and PRYGB cohorts showed a significant improvement from baseline in overall associated medical conditions after 2 years following RYGB (*p* < 0.001). The improvement of associated medical problems was defined according to the international guidelines [29]. Statistically significant improvements in diabetes mellitus (DM), ischemic heart diseases, hypertension, and dyslipidemia were recorded in both cohorts. Sleep apnea and hypertension were significantly improved in the RRYGB cohort. Renal insufficiency was not significantly impacted in both cohorts. (Table 4).

**Table 4:** Comparison of associated medical conditions of patients performing RRYGB post LAGB and patients performing PRYGB at preoperative, 1-, and 2-year follow-up

Comorbidities	Preoperative *n* (%)	At 1 year *n* (%)	At 2 years *n* (%)	Sig
Overall comorbid conditions				
RYGB post LAGB	46 (46.9) a	12 (12.2) b	4 (4.1) b, c	< 0.001*
Primary RYGB	43 (43.9) a	6 (6.1) b	2 (2.0) b, c	< 0.001*
IHD				
RYGB post LAGB	8 (8.2) a	3 (3.1) b	2 (2.0) b, c	0.006*
Primary RYGB	11 (11.2) a	2 (2.0) b	1 (1.0) b, c	< 0.001*
Sleep apnea				
RYGB post LAGB	7 (7.1) a	1 (1.0) b	0 b, c	0.002*
Primary RYGB	5 (5.1)	2 (2.0)	0	0.066
Dyslipidemia				
RYGB post LAGB	35 (35.7) a	3 (3.1) b	1 (1.0) b, c	< 0.001*
Primary RYGB	34 (34.7) a	1 (1.0) b	0 b, c	< 0.001*
Diabetes mellitus				
RYGB post LAGB	4 (4.1) a	0 b	0 b, c	0.018*
Primary RYGB	11 (11.2) a	0 b	0 b,c	< 0.001*
Hypertension				
RYGB post LAGB	9 (9.2) a	2 (2.0) b	1 (1.0) c	< 0.001*
Primary RYGB	6 (6.1) a	3 (3.1) ab	2 (2.0) b	0.039*
Renal insufficiency				
RYGB post LAGB	3 (3.1)	2 (2.0)	2 (2.0)	0.717
Primary RYGB	0	1 (1.0)	1 (1.0)	0.368

Related-Samples Cochran’s *Q* test compares proportion of positive complications per each surgery intervention with adjusted pairwise comparison. Significant pairwise comparison denoted by different superscripts. Percentage out of total patients undergoing RYGB post LAGB intervention (*n* = 98 after PSM) and Primary RYGB intervention (*n* = 98 after PSM)

*p ≤ 0.05

## Discussion

This was a retrospective study with a propensity score matching analysis that compared the outcomes between the one-stage RRYGB after LAGB and PRYGB during 2 years of follow-up.

### Revision surgery

Revision surgery continues to be an important division of bariatric surgery as many previously popular bariatric procedures proved inefficient regarding weight loss or had a high rate of complications. Revision bariatric procedures have increased over the last decade, accounting for 16.7% of all bariatric surgeries performed in the USA in 2019, increasing from 6% in 2011 [2]. Removal of adjustable gastric bands alone was formed 27.6% of all revision surgeries in 2018 [30].

Because LAGB is a reversible procedure, it is readily amenable to conversion to almost all known bariatric procedures. In our experience, we prefer the one- stage conversion of LAGB to RYGB. Conversion to RYGB has been advocated by many authors to be safe and effective for insufficient weight loss and band after LAGB [4, 11–16]. Also, data from systematic reviews have endorsed RRYGB for failed LAGB, and reported better and more sustained weight loss, and lower reoperation rates after RRYGB [7, 19].

Conversion of LAGB to RYGB or other bariatric procedures in one- or two-stage has always been a matter of debate. Traditionally, a two-stage procedure would give a chance to the foreign body reaction to subside and allow the definitive procedure to be done on less inflamed gastric tissue with less complications [30]. A one-stage procedure should lower cost, hospital stay, and peri-operative risk. However, the complex procedure of removing the band capsule to restore normal gastric tissue with division of adhesions and fibrous tissue, followed by the definitive bariatric procedure may be associated with more morbidity and longer operative time [4, 15, 17]. However, data from a recent meta-analysis reported a “safety advantage” for single-stage over two-stage RRYGB after LAGB [21]. Some authors recommended one-stage revisional surgery for LAGB in case of weight regain and two-stage procedure for band complications like erosion and infection [17, 23]. Weight loss was reported to be equal in one- or two- stage RRYGB [4, 23]. Optimizing safety must be the main goal if no difference is found in weight loss between one- and two- stage RRYGB.

RRYGB after LAGB is more difficult and demanding than primary procedures, with reported longer operative times [17, 31, 32]. The mean operative time in this study was significantly longer in RRYGB than in PRYGB (159.59 ± 34.3 min vs. 42.04 ± 9.2 min, respectively; *p* < 0.001), which agrees with the published data. Also, a two-stage RRYGB has a reported non-significant difference in operative time of the definitive procedure when compared to one-stage RRYGB (206.3 ± 73.5 vs. 208.5 ± 61.2 min, respectively) [4].

The main indication of one-stage RRYGB after LAGB is failed weight loss with a reported incidence of 61.6% [23]. Authors recommended one-stage RRYGB after LAGB for weight regain and two-stage RRYGB for band complications like erosion and infection [17, 23]. The main cause of revision in our study was weight regain and insufficient weight loss (87.6%).

### Weight loss

Both PRYGB and RRYGB cohorts exhibited significant reduction in BMI after 2 years compared to the baseline values before RYGB (*p* < 0.001), while the PRYGB cohort had significantly greater reduction in average BMI and increased %EBMIL during the follow-up period (*p* < 0.001). A reported probable cause for less weight loss with RRYGB is the possibility of a bigger pouch and less restriction when the surgeon avoids the scarred tissue while creating the gastric pouch; with reported estimated pouch volume of 60–80 cc [13]. Some authors reported less weight loss in the RRYGB after LAGB than PRRYGB, while other authors reported equal weight loss [31, 32] Data from a more recent meta-analysis reported 20% less weight reduction after RRYGB compared to that of PRYGB [33]. This suboptimal weight loss is inherent in the revisional procedures. Data from meta-analyses comparing the weight loss between RRYGB and revisional LSG (RLSG) for failed LAGB reported equal or greater weight reduction following RRYGB [18, 20]. Therefore, appropriate guidance, extra attention, and alternative therapy for the revision group postoperatively are necessary.

### Food tolerance (FT)

The average FT score in the RRYGB cohort displayed a significant increase in the first and second years of follow-up compared to the baseline (*p* < 0.001). This improvement is natural as LAGB is a totally restrictive operation with reported FT scores as low as 15.5 [34]. The release of restriction of the pouch, with improved eating, leads to increased FT score and possibly lower weight loss.

The PRYGB cohort had a significantly worse FT score compared to that of RRYGB in the first and second years of follow-up (*p* =  < 0.001 and *p* = 0.041, respectively). PRYGB reported low FT scores after surgery primarily due to dumping that improves with time [34, 35], and the new restriction with the new pouch. For both groups, extra attention on FT is required regarding RRYGB and the new eating pattern against weight gain and eating behavior with the new stomach size in PRYGB.

### Complications

In this study, the incidence of early and late complications was not significantly different in the primary and RRYGB cohorts. Authors reported no significant differences in the rates of complications between RRYGB after LAGB and PRYGB with rates ranging from 8.6% to 15.2% for RRYGB and from 5.5 to 14.7% for PRYGB [31, 32]. Data from large multicenter database analysis reported no significant difference in complications rates between one- and two-stage RRYGB after LAGB (13.5% vs. 10.8%, respectively) [15]. Data from meta-analyses reported; significantly higher rates of complications with RRYGB compared to PRYGB (18.6% vs. 8.6% respectively) [33]; overall complications rates of 8.3% and 8.9% for the one- and two-stage RRYGB after LAGB, respectively, and rates of 10.9% and 11.2% for one and two-stage RLSG after LAGB, respectively, with no significant difference [23]; and non-significant difference in the incidence of early and late complications between one- and two-stage RLSG and RRYGB after failed LAGB [18]. Also, some authors reported significantly higher overall early complications rates among patients who had one-stage RRYGB after LAGB than patients who had one-stage RLSG after LAGB (6.5% vs. 2.9%) [36]; while data from systematic reviews show no significant differences in complication rates between RRYGB and RLSG after LAGB [20]. Therefore, we can conclude that the one-stage RRYGB is a safe procedure to be performed.

This study had no incidence of the leak, while significant intra-abdominal bleeding occurred in 2 (2%) patients in the RRYGB cohort vs. 0% in the PRYGB cohort that required laparoscopic exploration. Leak and bleeding are well-known complications after RYGB with many authors reporting no significant differences in the incidence between primary and revision procedures and even between one- and two-stage revisions [4, 12, 13, 15, 17, 20, 23].

In this study, PMSVT was recorded in three (3.1%) of the RRYGB patients and in one (1%) PRYGB patient, however, all patients received prophylactic anticoagulation. Two of the four patients had a history of oral contraceptive pills (OCP) use, one had a history of preoperative anticoagulant use, and the 4th case had no obvious related risk factor. PMSVT is an uncommon complication after bariatric surgery with reported probable fatal outcomes due to bowel gangrene [37]. In general, PMSVT constitutes 5–15% of mesenteric ischemic events [38]. A rate of 0.3% for PMSVT following bariatric surgery was reported in meta-analyses, with active smoking and use of OCP identified as risk factors [37].

Intestinal obstruction due to internal hernia through the mesenteric defect at the jejuno-jejunostomy was recorded in one (1%) patient in RRYGB, despite routine closure of mesenteric defects with non-absorbable barbed sutures. Internal hernia can still occur with primary closure of the mesenteric defects with significantly the same low incidence rates compared to non-closure of the mesenteric defects [39, 40].

Marginal ulcers were recorded in 8 patients, 4 (4.1%) patients in each cohort, all were active smokers and 2 were also on NSAIDs. Medical treatment was successful in all patients. Our findings agree with the incidence of marginal ulcers in the literature (1–16%) with active smoking and NSAIDs as common risk factors and with successful medical treatment in 66–91% of patients [41].

Overall re-intervention rates in this study were 5.1 and 3.1% for the RRYGB and PRYGB cohorts, respectively (*p* = 0.721). Causes of re-intervention included bleeding, PMSVT, internal hernia, and port site hernia. Data from high-volume centers reported lower rates of re-intervention.

Data from high volume centers reported as low re-intervention rates of 1.1% in RRYGB after LAGB, which primarily included bleeding, and trocar site hernias [12]. Data from systematic reviews reported a higher rate of re-intervention after RRYGB than that of PRYGB (6.5 vs. 3.2%, respectively); causes of reoperation included intestinal obstruction, hiatal hernia, and anastomotic stenosis [17], which was consistent with our data.

### Associated medical problems

The outcomes were comparable in the PRYGB and RRYGB cohorts and both achieved a significant improvement in associated medical problems after 2 years compared to baseline before RYGB, (*p* < 0.001). The rates of resolution/improvement of type 2 DM were 100% in both RRYGB and PRYGB cohorts, 88.9 and 66.7% for HTN in RRYGB and PRYGB cohorts, respectively.

Data from meta-analyses reported non-significant differences in resolution/improvement rates of DM and HTN between PRYGB and RRYGB and reported pooled rates of remission and improvement of DM, HTN and sleep apnea of 46.5–84.0%, 35.9–71.6%, and 80.8% respectively [20, 33]. Nevertheless, even with a lower %EWL as one outcome marker, associated medical problems are possible more important for the physical expression of the patients. Therefore, revision surgery provided additional health benefits that need to be discussed when a patient is consulting a physician.

### Limitations

Propensity scores (PS) were developed to address bias in observational research. In the absence of randomization, the PS is a tool for creating matched pairs that balance the numerous observed covariables. Nevertheless, the potential limitation may be the lack of balance of residual confounders [42].

The retrospective design may be a point of weakness, as retrospective studies are inherently inferior to the prospective randomized trials that avoid the selection bias. However, there is no role for randomization in this study since a band is already in place.

PSM produced a smaller sample size that could potentially miss important findings in the patients excluded from the study.

## Conclusion

One-stage RRYGB for failed LAGB is a safe and efficient procedure with comparable rates of complications, re-interventions, and resolution of associated medical conditions to PRYGB. Weight loss following one-stage RRYGB was significantly lower than that of PRYGB but was still significant and satisfactory. FT improved significantly in the RRYGB patients after the revision procedure and was even significantly better than PRYGB patients.

## Appendix 1: Demographic and clinical characteristics of patients who underwent RRYGB

**Table:**

	Mean ± SD
Weight pre-band (kg)	139.59 ± 24.53
Least weight after band (kg)	98.51 ± 18.48
BMI pre-band	50.14 ± 7.88
Least BMI after band	35.41 ± 5.95
Age at band	33.31 ± 10.77
Time between band and RYGB (Years)	9.49 ± 9.01

	Frequency (%)
Reasons of revision	
Weight regain	73 (74.4)
Insufficient weight loss	13 (13.2)
Dysphagia	8 (8.2)
Failure of device	4 (4.08)

Endoscopy pre-RYGB	
Band erosion	1 (1.02)
Tight band	12 (12.24)
Reflux	9 (9.18)
Grade A	7 (7.14)
Grade B	1 (1.02)
Grade C	1 (1.02)
Hiatal Hernia	5 (5.10)
Dilated esophagus	14 (14.28)
